# The mismatch negativity: A review of underlying mechanisms

**DOI:** 10.1016/j.clinph.2008.11.029

**Published:** 2009-03

**Authors:** Marta I. Garrido, James M. Kilner, Klaas E. Stephan, Karl J. Friston

**Affiliations:** Wellcome Trust Centre for Neuroimaging, University College London, UK

**Keywords:** Mismatch negativity (MMN), Event-related potential (ERP), Mechanistic models, Cortical networks, Predictive coding

## Abstract

The mismatch negativity (MMN) is a brain response to violations of a rule, established by a sequence of sensory stimuli (typically in the auditory domain) [Näätänen R. Attention and brain function. Hillsdale, NJ: Lawrence Erlbaum; 1992]. The MMN reflects the brain’s ability to perform automatic comparisons between consecutive stimuli and provides an electrophysiological index of sensory learning and perceptual accuracy. Although the MMN has been studied extensively, the neurophysiological mechanisms underlying the MMN are not well understood. Several hypotheses have been put forward to explain the generation of the MMN; amongst these accounts, the “*adaptation hypothesis*” and the “*model adjustment hypothesis*” have received the most attention. This paper presents a review of studies that focus on neuronal mechanisms underlying the MMN generation, discusses the two major explanatory hypotheses, and proposes predictive coding as a general framework that attempts to unify both.

## 1 The MMN: a brief introduction

Small changes in the acoustic environment engage an automatic auditory-change detection mechanism reflected in the mismatch negativity (MMN). The presentation of an *oddball* or *deviant* event, embedded in a stream of repeated or familiar events, the *standards*, results in an evoked response that can be recorded non-invasively with electrophysiological techniques such as electro-encephalography (EEG) and magneto-encephalography (MEG). The MMN is the negative component of the waveform obtained by subtracting the event-related response to the *standard* event from the response to the *deviant* event. This brain response is measured with EEG and has a magnetic counterpart called MMNm. The MMN is elicited by sudden changes in stimulation, peaks at about 100–250 ms from change onset and exhibits the strongest intensity in temporal and frontal areas of topographic scalp maps (Sams et al., 1985). Given its automatic nature, the MMN might be associated with pre-attentive cognitive operations in audition and, for this reason, it has been suggested that it reflects ‘primitive intelligence’ in the auditory cortex (Näätänen et al., 2001). Here we finesse this notion and suggest that the mechanisms behind the generation of the MMN can be understood within a predictive coding framework that appeals to empirical Bayes.

While the MMN has been studied intensively in the auditory modality (for a recent review see Näätänen et al., 2007), some studies show evidence for the existence of a visual MMN counterpart (Astikainen et al., 2004; Czigler et al., 2004; see Pazo-Alvarez et al., 2003, for review). Omitted stimuli or deviances, such as direction of movement, form, orientation, location, contrast, size, spatial frequency and colour, elicit a negative component in the N2 latency range (250–450 ms). Nevertheless, there is controversy as to whether these N2-like waves elicited by visual stimulus change reveal the same degree of automaticity as in the auditory MMN or whether the emergence of this component is really based on a memory comparison process. A potential analogue to the MMN has also been reported in the somatosensory system, which seems to be generated in fine discrimination tasks (Kekoni et al., 1997; Akatsuka et al., 2005). Numerous studies have focused on event-related potential (ERP) scalp-maps, especially in clinical applications, when comparing, for instance, schizophrenic patients (Umbricht et al., 2003a) or dyslexic subjects (Baldeweg et al., 1999) with normal controls. The MMN has also been proved useful in understanding auditory perception and formation of sensory memory representations (Atienza et al., 2002; van Zuijen et al., 2006).

A major area of the MMN research is concerned with its underlying neuronal mechanisms. Several competing hypotheses have been put forward, based on experimental results obtained by ERPs, MEG and functional magnetic resonance imaging (fMRI). The most common interpretation is that the MMN arises whenever there is a break of regularity in a structured auditory sequence (Näätänen, 1992), and that a temporo-prefrontal network, comparing the current sensory input with a memory trace of previous stimuli, is responsible for generating this response at the scalp level (Giard et al., 1990; Rinne et al., 2000; Opitz et al., 2002; Doeller et al., 2003). From this perspective, the MMN is assumed to reflect an automatic auditory change detection process that triggers a switch in the focus of attention (Escera et al., 1998, 2003). However, this notion has been challenged recently by claims that the MMN rests on a much simpler mechanism, namely *neuronal adaptation* in the auditory cortex. The adaptation hypothesis proposes that the apparent MMN results from the subtraction of a N1 response to a novel sound, from the N1 response to a non-novel or repeated sound; where the N1 to a repeated sound is delayed and suppressed, as novelty decreases (Jääskeläinen et al., 2004).

In this paper, we review a variety of studies that have contributed to a mechanistic understanding of how the auditory MMN is generated, discuss the major hypotheses, and suggest a general and unifying framework; predictive coding, for understanding the MMN. Predictive coding is a general theory of perceptual inference. Under predictive coding the brain is regarded as a hierarchically organised cortical system, in which each level strives to attain a compromise between bottom-up information about sensory inputs, provided by the level below and top-down predictions (or priors) provided by the level above (Mumford, 1992; Rao and Ballard, 1999; Friston, 2003). Within this framework the MMN would result from a failure to predict bottom-up input and consequently to suppress prediction error (Friston, 2005; Baldeweg, 2006; Garrido et al., 2007). The predictive coding account of the MMN unifies the competing hypotheses of *neuronal adaptation* and *model adjustment* (Garrido et al., 2008).

Critically, predictive coding may rest on NMDA-dependent synaptic plasticity and its regulation by neuromodulatory transmitters (Friston, 2005). Pharmacological studies with substances that affect synaptic plasticity (using either direct NMDA [ant] agonists or drugs affecting neuromodulatory transmitter receptors) may therefore play an important role in investigating the neurobiological mechanisms underlying the MMN. Similarly, predictive coding may link clinical MMN studies to aberrant perceptual learning and NMDA-dependent synaptic plasticity. Given this, we include a brief overview of MMN changes in pharmacological and clinical studies. This serves as a prelude to the focus of this paper; predictive coding and the MMN.

## 2 General characteristics of the MMN

### 2.1 Scalp topography

**Fig. 1:**
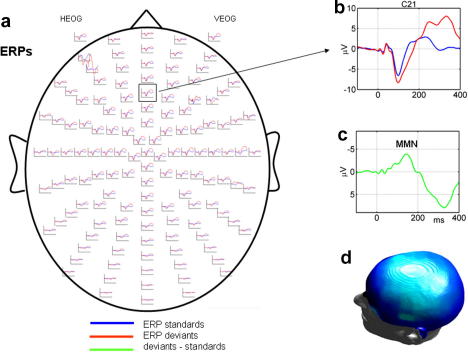
Scalp topography and time latency of the MMN. (a) ERP responses to standard and deviant tones overlaid on a whole scalp map of 128 EEG electrodes. (b) ERP responses to the standard and deviant tones at a fronto-central channel. (c) MMN difference wave obtained by subtracting ERP to standards from ERP to deviants. (d) MMN response averaged over the time window of 100–200 ms interpolated for a 3D scalp topography. (From Garrido et al., 2007.)

The MMN is the negative component of a difference wave between responses to standard and deviant events embedded in an *oddball* paradigm. This negative response, of about 5 μV maximum peak, is distributed over fronto-central scalp locations (see Fig. 1).

The MMN peaks at about 100–250 ms after change onset but this latency varies slightly according to the specific paradigm or the type of regularity that is violated: frequency, duration, intensity, or the inter-stimulus interval (Näätänen et al., 2004) (see Fig. 1c). In more complex paradigms an abstract rule is broken, such as inter-stimulus relationships (Tervaniemi et al., 1994; Paavilainen et al., 2001; Vuust et al., 2005) or phoneme regularity (Näätänen et al., 1997). Barely discriminable tones elicit a later MMN peaking at about 200–300 ms (Näätänen and Alho, 1995).

### 2.2 MMN under different paradigms

The MMN is elicited in the presence of any discriminable change in some repetitive aspect of auditory stimulation. This discriminable change can be of different types: frequency, duration, intensity, perceived sound-source location, silent gap instead of a tone, or one phoneme replaced by another. In a recent study, Näätänen et al. (2004) proposed a new paradigm, in which a standard alternates with one of five deviant types that differ in duration, location, intensity, gap and frequency. Because of its effectiveness, this paradigm is particularly useful in clinical research as it can be used to obtain five different types of MMN responses in the same time traditional paradigms elicit only one type of MMN.

It is generally believed that the MMN is evoked by any violation of an acoustic regularity or pattern. Indeed, the MMN is elicited by violations of abstract rules established in a structured auditory sequence (Näätänen et al., 2001). For example, with complex auditory patterns, it has been found that an MMN is elicited by an occasional ascending tone or tone repetition in a sequence of regularly descending tone pairs (Tervaniemi et al., 1994); by changing the direction of within-pair frequency change (Saarinen et al., 1992); independently of their absolute frequencies, and by violations of the rule that the higher the frequency, the louder the intensity (Paavilainen et al., 2001). The MMN is also detected when the stimuli are spectrally rich. This type of paradigm facilitates attentive pitch discrimination in comparison to pure sinusoidal tones; in other words, the MMN is larger and has shorter latency (Tervaniemi et al., 2000a). Moreover, MMN responses are elicited by breaking a regularity in roving paradigms (Baldeweg et al., 2004; Haenschel et al., 2005; Garrido et al., 2008), or in more sophisticated paradigms comprising irregularities in rhythms (Vuust et al., 2005), musical sequences (van Zuijen et al., 2004), and violations in phoneme regularity (Näätänen et al., 1997).

### 2.3 An index of memory traces?

It is commonly accepted that the MMN rests on the relation between the present and the previous stimulus, rather than on the stimulus alone. Hence, the MMN may depend on a memory trace formed by preceding stimuli; i.e., during the presentation of the *standard* events. If the *deviant*, or the *new* event, occurs while this memory trace is still active, the automatic change-detection is activated, giving rise to a MMN response (Näätänen, 2000). The duration of this period, also called echoic memory, has been reported to last at least 10 s in normal subjects (Böttcher-Gandor and Ullperger, 1992).

### 2.4 Dependence on attention?

The MMN is the earliest ontological cognitive component that can be observed in an ERP trace (Alho et al., 1990). An important characteristic of the MMN in auditory *oddball* paradigms is the fact that it can be detected even when the subject is not paying attention. The MMN can be measured without any task requirements and is elicited even when the subject performs a task that is not related to the stimulus. The MMN can be elicited irrespective of attention, during non-attentive states such as sleep (Sallinen et al., 1994), or even in coma; where the presence of a MMN has been proposed as a predictor for recovery of consciousness (Kane et al., 1993). This demonstrates the brain’s capacity to perform complex comparisons between consecutive sounds automatically (Näätänen et al., 2001). Although the MMN is seldom affected by attention, some studies suggest that the MMN is attenuated when the subject’s attention is outside the focus of the auditory stimulus (Arnott and Allan, 2002; Müller et al., 2002). On the other hand, the degree to which the visual stimulus is attended does not seem to influence the MMN (Otten et al., 2000). To avoid overlap with other ERP components, some authors argue that the best condition to observe an MMN is when subject attention is directed away from the stimulus (Näätänen, 2000).

It has been reported that the generation of the MMN, in particularly the source over the frontal lobe, is associated with an involuntary attention switching process, an automatic orienting response to an acoustic change (Escera et al., 1998, 2003). In addition, it has been suggested that the frontal generator of the MMN is related to an involuntary amplification or contrast enhancement mechanism that tunes the auditory change detection system (Opitz et al., 2002).

## 3 The relevance of the MMN and its applications

The fact that MMN can be elicited without special task requirements, independently of the subject’s motivation and in the absence of attention, during sleep, or even before coma recovery, makes it particularly suitable for testing different clinical populations, infants and newborns (see Kujala et al., 2007, for a recent review). The following two subsections present a brief review of recent studies that used the MMN to address important questions in cognitive processing and clinical neuroscience.

### 3.1 MMN in cognitive studies

The MMN is considered to represent the only objective marker for auditory sensory accuracy (Näätänen, 2000). MMN studies have made important contributions to our understanding of the formation of auditory perception and streaming (see Denham and Winkler, 2006, for a review), construction of sensory memory representations, as well as how these are influenced by attention (Sussman et al., 1998; Sussman and Steinschneider, 2006). It has been shown that whereas attention is not always necessary for auditory stream segregation (Sussman et al., 2007), switches in attention are important for streaming reset (Cusack et al., 2004). Woldorff et al. (1993) have shown that focused auditory attention can modulate sensory processing as early as 20 ms. Others have used the MMN to characterise the mechanisms of involuntary attention switching (Escera et al., 1998, 2003).

Several studies have used the MMN to understand mechanisms of perceptual learning. Tremblay et al. (1998) showed that training-associated changes in neural activity, indicated by the MMN, precede behavioural discrimination of speech. The MMN was also found to correlate with gains in auditory discrimination after sleep (Atienza et al., 2002, 2005). Implicit, intuitive and explicit knowledge have been characterised in terms of the elicited responses, the MMN and P3, combined with behavioural measures (van Zuijen et al., 2006).

### 3.2 MMN in clinical neuroscience

The MMN has proved useful in various clinical contexts (see Näätänen, 2000, 2003 for reviews on clinical research and applications). The most promising clinical application of MMN is in schizophrenia research. More than 30 studies have found significant reductions of MMN amplitude in patients with schizophrenia, both for frequency and duration deviants (Umbricht and Krljes, 2005). Moreover, individual MMN amplitudes correlate with disease severity and cognitive dysfunction (Baldeweg et al., 2004) and functional status (Light and Braff, 2005), although there are conflicting reports about its association with genetic risk for schizophrenia (Michie et al., 2002; Bramon et al., 2004). Two features make the MMN a particularly interesting paradigm for schizophrenia research (see Stephan et al., 2006, for a review). First, the MMN depends on intact NMDA receptor signalling: pharmacological blockage of NMDA receptors has been shown to significantly reduce the MMN, both in invasive recordings studies in monkeys (Javitt et al., 1996) and human EEG/MEG studies (Kreitschmann-Andermahr et al., 2001; Umbricht et al., 2000, 2002). This is important because the critical role of the NMDA receptor in the plasticity of glutamatergic synapses is at the core of current pathophysiological theories of schizophrenia (Friston and Frith, 1995; Harrison and Weinberger, 2005; Javitt, 2004; Stephan et al., 2006). Second, clinical investigations of schizophrenic patients require very simple paradigms that are robust to changes in attention and performance. As discussed above, the MMN fulfils these requirements very well.

The MMN has proved useful for investigating several diseases in addition to schizophrenia. Another important application is in the field of dyslexia: dyslexic patients show diminished MMN, albeit only for frequency deviants and not for duration. This suggests that dyslexia is associated with auditory frequency discrimination impairment (Baldeweg et al., 1999). A reduced MMN in children with learning disabilities suggested that the deficit originates in the auditory pathway at a processing stage prior to conscious perception (Kraus et al., 1996). This is in accord with Rinne et al. (1999) who showed that speech processing occurs at early pre-attentive stages on the left hemisphere (at about 100–150 ms after sound onset).

### 3.3 The MMN and neuropharmacology

Pharmacologically induced changes in the MMN have been investigated in numerous studies, using a variety of drugs affecting different neurotransmitter systems. The most robust, and perhaps also the most important neuropharmacological effect, given its importance for relating the MMN to schizophrenia, is exerted through NMDA receptors: several studies have found strong reductions of MMN amplitude under the NMDA antagonist ketamine (Ehrlichman et al., 2008; Heekeren et al., 2008; Javitt et al., 1996; Kreitschmann-Andermahr et al., 2001; Umbricht et al., 2000, 2002), with only a single study failing to find a significant effect of ketamine (Oranje et al., 2002).

In contrast to NMDA receptors, the roles of dopamine, serotonin, nicotinic, muscarinic and GABA receptors for MMN generation are more controversial. With regard to dopamine; early studies reported a decrease of MMN amplitude in patients with Parkinson’s disease (Pekkonen et al., 1995) and in healthy volunteers treated with the D2-receptor antagonist haloperidol (Kähkönen et al., 2001). A subsequent combined MEG/EEG study of healthy volunteers receiving haloperidol could not replicate this result, but only found a shorter latency of the MMN that was specific for MEG measurements (Pekkonen et al., 2002). Similarly, a recent study using both D1- and the D2-receptor agonists found no evidence for MMN modulation by dopaminergic receptors (Leung et al., 2007). Data on the relation of serotonin receptors to MMN generation are similarly inconsistent. Kähkönen et al. (2005) used acute tryptophan depletion in healthy volunteers to reduce serotonin synthesis in the brain; they found significantly reduced MMN amplitude and a shortened latency. In contrast, an EEG study in healthy volunteers, using the 5HT2A receptor antagonist psylocibin, found no evidence of MMN modulation (Umbricht et al., 2003b). Concerning nicotinic receptors, the literature is less diverse, albeit not fully consistent; whereas most studies reported an increase in the MMN amplitude by nicotinic receptor stimulation (Baldeweg et al., 2006; Dunbar et al., 2007; Engeland et al., 2002), other studies found nictonergic effects on latency and width of the MMN (Harkrider and Hedrick, 2005; Inami et al., 2005), and one study did not find any effect at all (Knott et al., 2006). The only two available studies on the role of muscarinic receptors in the MMN, performed by the same authors, gave contradictory results (Pekkonen et al., 2001, 2005). Finally, inconsistent results have also been obtained in studies manipulating GABA_A_ receptor function, with some studies reporting a significant reduction of MMN amplitude by benzodiazepines (Nakagome et al., 1998; Rosburg et al., 2004), whereas other studies failed to observe a significant modulation of the MMN (Kasai et al., 2002; Murakami et al., 2002; Smolnik et al., 1998).

Overall, one might conclude that the roles of dopaminergic, serotoninergic, muscarinic and GABA receptors in MMN generation are currently not well established and require further research. The evidence for an involvement of nicotinic receptors is stronger, albeit not fully consistent. In contrast, there is broad agreement amongst studies that blockage of NMDA receptors leads to significant reductions in MMN amplitude.

## 4 The mechanisms of MMN generation

Despite the vast literature on MMN research, the mechanisms that underlie its generation remain a matter of debate. Two major competing hypotheses have emerged, the *model adjustment hypothesis* and the *adaptation hypothesis*. The following subsections describe these two competing hypotheses and discuss the experimental evidence that favours one or the other. Finally, predictive coding is suggested as a unifying framework that can accommodate both hypotheses. This idea is supported by recent results from our connectivity modelling approach to the MMN (Garrido et al., 2008, under review).

### 4.1 The model adjustment hypothesis

**Fig. 2:**
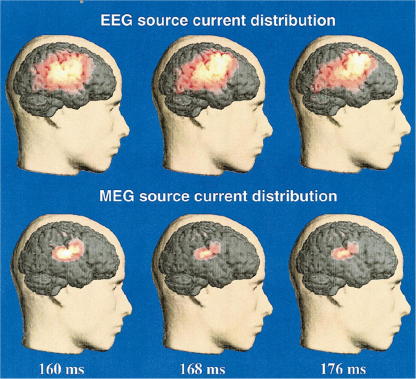
MMN generators estimated from EEG and MEG data. The centre of gravity changes from temporal to frontal areas over time. Frontal sources were detected with EEG; due to their radial orientation they might not be detected by MEG. These sources were determined with minimum norm estimates (MNE). (Adapted from Rinne et al., 2000.)

The MMN can be regarded as an index of automatic change-detection governed by a pre-attentive sensory memory mechanism (Tiitinen et al., 1994). Several studies have proposed mechanistic accounts of how the MMN might be generated. The most common interpretation is that the MMN is a marker for error detection caused by a break in a learned regularity or familiar auditory context. The MMN would then result from the difference, or mismatch, between the current and preceding input. Early work by Näätänen and colleagues suggested that the MMN results from a comparison between the present auditory input and the memory trace of previous sounds (Näätänen, 1992). In agreement with this theory, others (Winkler et al., 1996; Näätänen and Winkler, 1999; Sussman and Winkler, 2001) have postulated that the MMN could reflect on-line modifications of a perceptual model that is updated when the auditory input does not match its predictions. This is the so-called *model-adjustment hypothesis*. In the context of the model adjustment hypothesis, the MMN is regarded as a marker for error detection, caused by a deviation from a learned regularity. In other words, the MMN results from a comparison between the auditory input and a memory trace of previous sounds, reflecting an on-line updating of the model for predicting auditory inputs (Winkler et al., 1996; Näätänen and Winkler, 1999). According to this hypothesis, the MMN is a response to an unexpected stimulus change. This hypothesis has been supported by Escera et al. (2003) who provided evidence for the involvement of the prefrontal cortex in providing top-down modulation of the deviance detection system in the temporal cortices. In the light of Näätänen’s model, it has been claimed that the MMN is caused by two underlying functional processes, a sensory memory mechanism related to temporal generators and an automatic attention-switching process related to the frontal generators (Giard et al., 1990). The role of prefrontal generators is supported by studies of patients with prefrontal lesions who showed diminished temporal MMN amplitudes (Alain et al., 1998). Furthermore, it has been shown that the temporal and frontal MMN sources have separate temporal dynamics (Rinne et al., 2000) but interact with each other (Jemel et al., 2002). This notion is also compatible with strong and reciprocal anatomical connectivity between auditory and prefrontal areas that has been found in primate tract tracing studies (Romanski et al., 1999). According to source reconstruction studies, the generators of the MMN are located bilaterally in the temporal cortex (Hari et al., 1984; Giard et al., 1990; Alho, 1995). In addition, there is evidence for generators in the prefrontal cortex, often stronger and reported more consistently on the right hemisphere for tone paradigms (Levänen et al., 1996) and on the left hemisphere for language paradigms (Näätänen et al., 1997; Tervaniemi et al., 2000b; Pulvermüller, 2001). A sensory memory mechanism has been associated with the temporal generators, whereas a cognitive role, or comparator-based mechanism, has been assigned to the prefrontal generators (Giard et al., 1990; Gomot et al., 2000; Maess et al., 2007). Numerous studies have consistently reported evidence for multiple generators of the MMN in the primary auditory cortex. This has been reproduced across different modalities such as EEG (Deouell et al., 1998; Jemel et al., 2002; Marco-Pallarés et al., 2005; Grau et al., 2007), MEG (see for example Tiitinen et al., 2006; or Hari et al., 1984) and combined EEG with MEG measures (Rinne et al., 2000). The latter study revealed that prefrontal generators are activated later than the generators in the auditory cortex; this supports the hypothesis of a change detection mechanism in the prefrontal cortex, which is triggered by the temporal cortex. This study found temporal sources with both M/EEG, whereas prefrontal sources were only found with EEG; possibly because the frontal sources have a radial orientation and the MEG sensors are blind to the fields generated by radial sources (see Fig. 2).

**Fig. 3:**
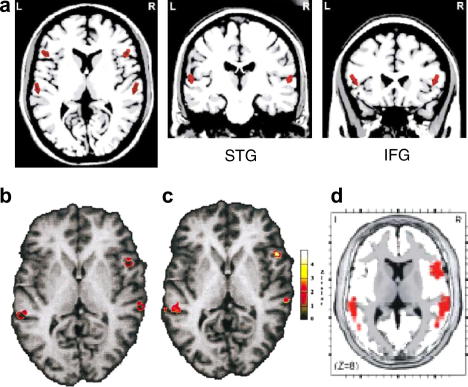
MMN underlying sources revealed by EEG and conjoint EEG and fMRI measures. (a) Dipoles indicated by red arrows at bilateral **STG** and **IFG** (adapted from Doeller et al., 2003). (b) Dipole locations at bilateral **STG** and right **IFG** and (c) significant fMRI activation for deviants (adapted from Opitz et al., 2002). (d) Most significant independent component (computed by ICA-LORETA analysis, adapted from Marco-Pallarés et al., 2005). This figure shows consistency for MMN sources across different modalities.

fMRI (Molholm et al., 2005; Rinne et al., 2005) and combined fMRI-EEG studies (Opitz et al., 2002; Doeller et al., 2003; Liebenthal et al., 2003) have reported findings that are consistent with the results of the source reconstruction studies described above. Some of the combined fMRI-EEG studies show a double peak over frontal scalp locations suggesting the existence of two subcomponents for the MMN. Dipole modelling was performed in two time windows to explain the scalp ERP distribution (Opitz et al., 2002; Doeller et al., 2003). The early component is reported to peak in the time window of 90–120 ms and it can be modelled with dipoles located bilaterally in the superior temporal gyrus (**STG**). ERPs within the late time window, 140–170 ms, can be modelled with dipoles placed in left and right inferior frontal gyrus (**IFG**) (see Fig. 3). The sources in the temporal areas might be involved in processing changes of the sound’s physical properties, whereas the sources on the frontal areas might reflect reorientation of attention. Recent work has linked the early component (in the range of about 100–140 ms) to a sensorial, or non-comparator account of the MMN, originated in the temporal cortex, and the later component (in the range of about 140–200 ms) to a comparator-based mechanism of the MMN, involving the prefrontal cortex (Maess et al., 2007). Although MMN sources are found consistently over temporal and pre-frontal regions, a few studies have reported sources at other locations such as right temporal and parietal lobes (Levänen et al., 1996).

Thus, these studies provide evidence that the MMN is generated by a temporo-frontal network, which appeals to the model adjustment hypothesis. This rests on a change-detection mechanism; in which the MMN reflects greater prediction error or mismatch between top-down predictions and current inputs. In other words, the MMN is elicited when there is a change in the input, relative to the predictions formed on the basis of a memory trace of previous input. Clearly, the implicit increase in prediction error signifies something has changed and calls for an adjustment of the brain’s internal model or memory of the stimulus.

### 4.2 The adaptation hypothesis

A recent study (Jääskeläinen et al., 2004) has challenged the common view that the MMN is generated by a temporal–frontal cortical network. Instead, they suggest that the MMN results from a much simpler mechanism of local neuronal *adaptation* at the level of the auditory cortex, causing attenuation and delay of the N1 response. The N1 response is the negative component peaking at about 100 ms from stimulus onset and is associated with early auditory processing at the level of A1. They propose that the N1 response to standard (or ‘non-novel’) sounds is delayed and suppressed (or *attenuated*) as a function of its similarity to the preceding auditory events, reflecting short-lived adaptation of auditory cortex neurons.^1^ As a consequence, the observed response would be erroneously interpreted as a separate component from the N1 wave. According to the *adaptation hypothesis*, the fact that the neuronal elements within the auditory cortex become less responsive during continuous stimulation is sufficient to explain the generation of an apparent MMN. With the generation of a delayed and suppressed N1 in the auditory cortex, the MMN would emerge as a product of an N1 differential wave when subtracting the ERP to the standards from the ERP to the deviant.

The adaptation hypothesis rests on previous MEG studies indicating the presence of two subcomponents of the N1 response: a posterior subcomponent, N1p, peaking at about 85 ms from stimulus onset, and an anterior subcomponent, N1a, peaking at about 150 ms (Loveless et al., 1996). The amplitude of the posterior component is strongly suppressed during the presentation of identical stimuli, whereas this adaptation effect is smaller for the anterior component. In contrast to previous studies showing that repetitive standard sounds constitute a prerequisite for the MMN, Jääskeläinen et al. (2004) furnish evidence for robust MMN to infrequent (or ‘novel’) stimuli when preceded by a single standard stimulus. Consistent with the *adaptation* hypothesis, EEG measurements employing small deviances around a standard tone demonstrate that the smaller the frequency separation between the standard and the deviant, the more the amplitude to the deviants is attenuated (May et al., 1999).

*Adaptation* is a compelling hypothesis for the generation of the MMN that explains the experimental results mentioned above. However, there are other empirical observations that are not compatible with the *adaptation* hypothesis (see Näätänen et al. (2005) for a critical assessment on the *adaptation* view of Jääskeläinen et al., 2004). One of the points against *adaptation* is the fact that it predicts that the MMN duration and latency should match those of the N1, which has been shown not to be the case (Winkler et al., 1997). Secondly, *adaptation* does not explain why an MMN can be elicited in the absence of a N1 response, for example, during sleep (Atienza and Cantero, 2001) or when unexpectedly omitting a stimulus (Yabe et al., 1997). However, one potential defence in favour of the adaptation hypothesis rests on the notion that neuronal dynamics, induced by rhythmic stimulation, continue to oscillate upon cessation or interruption of stimulation (May et al., 1999). A third and compelling piece of evidence is that infrequent decrements in tone intensity also evoke an MMN (Näätänen et al., 1989). A MMN to a reduced stimulus intensity (or indeed omission of a stimulus) is difficult to explain in terms of *adaptation* alone. Another point of controversy is that, as mentioned above, the violation of abstract rules or complex inter-stimulus relationships can also elicit an MMN. For instance, an ascending tone pair in a sequence of descending tone pairs elicits an MMN (Saarinen et al., 1992) even though there is no stimulus repetition that could cause adaptation of a frequency-specific neuronal population. Given the tonotopic structure of auditory cortex, MMNs of this sort cannot be explained by local adaptation but must result from more complex mechanisms involving more than one neuronal population. Moreover, the scalp distribution of the MMN is different from the N1 (Giard et al., 1990). While the N1 components are larger in amplitude over the contralateral hemisphere, the MMN response to changes in acoustical features is right-hemispheric dominant (Levänen et al., 1996) and left-hemispheric dominant for phoneme changes, irrespective of the ear stimulated (Näätänen et al., 1997). Recently, Horváth et al. (2008) used a refined oddball paradigm that minimises the N1 confound, to show that frequency deviations have an effect on the N1 component but do not influence the MMN proper. This supports the notion that *adaptation* contributes to the MMN (as measured in conventional paradigms, i.e., MMN confounded with the N1 component), but is not sufficient to explain the MMN *per se*. Another finding that cannot be explained by *adaptation* alone is that equivalent current dipole (ECD) modelling reveals that the temporal source underlying the MMN is located more anterior than the source underlying the N1 (Hari et al., 1992; Tiitinen et al., 1993). Besides that, the MMN has a second main source in the frontal lobe, which expresses temporal dynamics that are distinct from the N1 source (Opitz et al., 2002; Doeller et al., 2003; Molholm et al., 2005; Grau et al., 2007). Evidence for a frontal generator was also provided from direct intracranial recordings in human epilepsy patients (Rosburg et al., 2005). Finally, pharmacologic manipulations show that NMDA antagonists block the generation of MMN without affecting activity in the primary auditory cortex (Javitt et al., 1996), which suggests that the MMN and the N1 employ different neuronal populations and are expressions of separate cortical processes. Finally, if the MMN results from neuronal adaptation, one would predict changes in MMN following manipulations of serotoninergic and muscarinic receptors. This is because activation of these receptors is known to enhance neuronal adaptation (cf. McCormick and Williamson, 1989). As described above, however, there is only weak and contradictory empirical evidence for MMN modulation by serotoninergic and muscarinic agents.

### 4.3 The MMN from the perspective of predictive coding

**Fig. 4:**
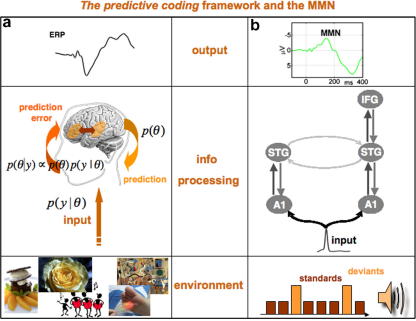
The MMN interpreted in terms of predictive coding. (a) Illustrative scheme of the general framework of hierarchical Bayes and predictive coding as an explanation for ERP emerge. (b) The MMN, a concrete example and plausible underlying mechanisms.

Predictive coding (or, more generally, hierarchical inference in the brain) states that perception arises from integrating sensory information from the environment and our predictions based on a model of what caused that sensory information. Prediction error is minimised through recurrent interactions amongst levels of a cortical hierarchy in order to estimate the most likely cause of the input (Friston, 2003, 2005). The model adjustment hypothesis explains the MMN as a marker for error detection caused by a deviation from a learned regularity. The MMN would thus result from a comparison between the auditory input and a memory trace embodied in top-down predictions. The ensuing prediction error may then be used for on-line updating of a model for predicting auditory inputs (Winkler et al., 1996; Näätänen and Winkler, 1999). This is completely consistent with the predictive coding framework, where current inputs are predicted from past inputs (see Fig. 4). In the case of a prediction error, i.e. when there is a mismatch between the predicted and the actual sensory input, the neural system implementing the model must be adjusted (for example, by short-term synaptic plasticity). During the repetition of subsequent events, that adjustment is reflected neurophysiologically in the suppression of prediction error and the disappearance of the MMN (Friston, 2005; Baldeweg, 2006). This view is identical to predictive coding models of vision, which postulate that perception relies on hierarchically organised neural systems, in which each level compares predictions from higher-level areas with information from lower areas (Rao and Ballard, 1999; Yuille and Kersten, 2006): using backward connections, higher cortical areas attempt to fit their abstractions, or learned reconstructions of the world, to the data received from lower cortical areas. The lower areas, in turn, attempt to reconcile the predictions from higher areas with the actual input, and return, by means of forward connections, a prediction error signal, i.e. information on the features not predicted by the higher areas (Mumford, 1992). Hence, lower and higher areas communicate via reciprocal pathways until reconciliation; in other words, until the prediction error is suppressed and the encoding of sensory causes at higher cortical areas is optimised (Friston, 2003).

Recently, predictive coding has been formulated in terms of empirical Bayesian models of perceptual learning and inference. The ensuing framework provides a nice way to understand the MMN (Friston, 2003, 2005; Garrido et al., 2008). In empirical or hierarchical Bayes, priors p(θ) about the underlying causes of sensory input, are optimised in higher hierarchical levels (i.e., cortical areas) and provide top-down constraints on the most likely representations in lower levels. These ‘most likely’ representations maximise the posterior or conditional density p(θ|y) of the causes of sensory data *y*. The conditional density is defined by Bayes rule

p(θ|y)∝p(θ)p(y|θ)

This rule combines the top-down prior and a likelihood, p(y|θ), which corresponds to the generative model used by the brain to predict its sensory input (see Fig. 4). In practice, this form of Bayesian inference can be implemented by message-passing amongst hierarchical levels of the cortex; where top-down predictions are passed to lower levels to explain away bottom-up inputs. The resulting prediction error is then passed back up the hierarchy, to optimise high-level representations. When the message-passing converges, the representations at all levels correspond to the conditional expectation of the causes of sensory input; i.e., a multilevel representation. This scheme provides a compelling model for evoked sensory responses, in terms of self-organised reciprocal exchanges between cortical areas to produce transients of neuronal activity. Put simply, neuronal activity tries to suppress prediction error to represent the states of the world; this is perceptual inference. However, over repeated presentations of the same stimulus, connection strengths that encode statistical regularities in the world also change to reduce prediction error. This corresponds to perceptual learning and is the mechanism we think underlies the MMN.

Critically, hierarchical inference (e.g., predictive coding) also rests on optimising the relative influence of bottom-up prediction error and prediction error based on top-down prior expectations. This involves optimising the post-synaptic sensitivity (and lateral interactions) of prediction error units within an area or source (Friston, 2003). Put simply, when a standard stimulus can be predicted more precisely by top-down afferents, less weight is assigned to bottom-up influences and the post-synaptic responsiveness to sensory inputs decreases. This is exactly what the adaptation hypothesis predicts. In short, hierarchical inference, using prediction error, provides a principled framework in which the model adjustment and adaptation heuristics become necessary for sensory inference.

We have seen that predictive coding formulations entail specific mechanisms that might underlie the MMN. A promising approach, to address these mechanisms, is to create biophysically realistic models that can represent competing hypotheses. These models can be tested empirically and provide evidence to disambiguate amongst competing theories. A pioneering study of this sort was performed by May et al. (1999) who constructed a model of tonotopically organised auditory cortex consisting of leaky integrate-and-fire neurons and compared its predictions to experimentally measured MEG/EEG data. Their question was whether the MMN could be explained by a local post-synaptic mechanism (i.e., neuronal adaptation) alone, or whether additional non-local synaptic mechanisms were required. They chose lateral inhibition (i.e., reciprocal inhibitory connections amongst neighbouring neuronal populations) as a candidate mechanism of the latter sort. They found that their experimental data could best be approximated by a model that combined adaptation and lateral inhibition.

Another class of models are those that use dynamic causal modelling (DCM) to test the likelihood of plausible connectivity graphs underlying the MMN, and to infer the coupling parameters of the most likely network. Dynamic causal modelling is a generic approach to modelling the neuronal mechanisms that underlie measured neuroimaging (Friston, 2003; Stephan et al., 2007; Marreiros et al., 2008) and electrophysiological data (David et al., 2006; Kiebel et al., 2006, 2007; Garrido et al., 2007). A recent study (Garrido et al., 2008) compared different accounts of the mechanisms underlying MMN generation, using DCMs for M/EEG data. DCM for electrophysiological data combines a neural mass model with a forward model that translates the neural dynamics into predicted measurements; estimation techniques based on a variational Bayes allow one to infer the parameters of the neuronal system from the observed data. In the study by Garrido et al. (2008), Bayesian model comparison (Penny et al., 2004) was used to select the best amongst several DCMs that represented competing mechanistic hypotheses about MMN generation. The range of models tested included (i) the *adaptation* hypothesis, i.e. that the MMN is best explained by a deviant-induced suspension of neuronal adaptation that is confined to lower-order auditory areas (cf. Jääskeläinen et al., 2004); (ii) the *model-adjustment* hypothesis (Winkler et al., 1996; Doeller et al., 2003) which assumes that the MMN results from deviant-induced changes in temporo-frontal connections; i.e. short-term synaptic plasticity; and (iii) combinations of these two hypotheses which accommodate intra-areal adaptation combined with plasticity of inter-areal connections. The latter group of models are consistent with the *predictive coding* formulation. Our results suggest that the mechanisms of MMN generation involve plasticity in inter-areal connections amongst multiple hierarchical levels, as well as local adaptation within the primary auditory cortices. These results indicate that the adaptation hypothesis is not sufficient to explain MMN generation, nor do they favour model adjustment alone. In other words, the MMN cannot be explained by changes in post-synaptic sensitivity or intrinsic connections, only; nor can it be explained by exclusive changes in extrinsic connections. This result is important because it supports a model that combines both the *model adjustment hypothesis* (Winkler et al., 1996) and the local *adaptation* hypothesis (Jääskeläinen et al., 2004) into the unified and more general framework of predictive coding. Moreover it can accommodate the findings of a multitude of studies showing that there are temporal and frontal cortical sources underlying the MMN generation (Rinne et al., 2000; Jemel et al., 2002; Opitz et al., 2002; Doeller et al., 2003; Liebenthal et al., 2003; Molholm et al., 2005; Restuccia et al., 2005).

An example of experimental evidence that can be reinterpreted in terms of predictive coding is that dipole intensity is stronger for large deviants (100%) compared with medium deviants (30%) at the temporal sources (Opitz et al., 2002). On the other hand, a reversed pattern was observed in the right frontal cortex; i.e., a bigger dipole strength in case of low discrimination between a sensory memory trace and auditory input. The authors discuss these findings in terms of alternative explanations and suggest that the prefrontal cortex (**IFG**) contributes to a top-down process that modulates the deviance detection system in the temporal cortices (**STG**) (see also Doeller et al., 2003). Under Bayesian models of perception (Yuille and Kersten, 2006) this dissociation can be interpreted easily as greater prediction error in low level sources for large deviants. Conversely, in higher levels, ambiguous bottom-up cues may induce prediction errors that cannot be explained away by even higher levels. Very similar dissociations between high and low-level responses to predictable and unpredictable stimuli have bee reported in the visual cortex (e.g., Murray et al., 2004; Harrison et al., 2007).

In summary, the predictive coding framework postulates that evoked responses correspond to prediction error that is explained away (within trial) during perception and is suppressed (between trials) by changes in synaptic sensitivity and efficacy during perceptual learning. The predictive coding framework encompasses the two distinct hypotheses, in the sense that it predicts the adjustment of a generative model of current stimulus trains (cf. *the model-adjustment hypothesis*) by using plastic changes in synaptic connections (cf. *the adaptation hypothesis*). The repeated presentation of standards may render suppression of prediction error more efficient; leading to a reduction in evoked responses under repetition and the emergence of a mismatch response, when an unlearned stimulus is presented. In this framework, increases in intrinsic connectivity may encode progressive increases in the estimated precision of top-down predictions, responsible for suppressing prediction error. These changes could be mediated by adaptation-like mechanisms in the auditory cortices to repeated sounds. Changes in forward connections may reflect changes in sensitivity to prediction error that is conveyed to higher levels. These higher levels form predictions so that backward connections can provide contextual guidance to lower levels. In this view, the MMN represents a failure to predict bottom-up input and consequently a failure to suppress prediction error.

In conclusion, the predictive coding model provides a common framework for both *adaptation* and *model-adjustment*. This framework lends a probabilistic perspective to conventional views of the MMN. Moreover, predictive coding gracefully subsumes synaptic activity, sensitivity and plasticity within the same optimisation scheme. This is important because optimum inference requires both optimisation of neuronal representations (as reflected in the ERP *per se*) and changes in synaptic responsiveness and efficiency (as reflected in the MMN or ERP difference). Furthermore, it shows how change-detection, adaptation and model-adjustment can all be understood as aspects of the same perceptual optimisation. In short, predictive coding reconciles apparently distinct models of the MMN and affords a neurobiological mechanism for its generation, which embodies both *adaptation* and *model-adjustment*.

## Footnotes

1 Neuronal adaptation, or spike-frequency adaptation, results from activation of calcium-dependent potassium channels that lead to a slow after hyperpolarizing currents, decreasing neuronal excitability and firing rate (see Faber and Sah, 2003, for review). Adaptation is thus a local phenomenon that is independent of pre-synaptic mechanisms and rests on changes in post-synaptic responsiveness.
